# Food Traceability as an Element of Sustainable Consumption—Pandemic-Driven Changes in Consumer Attitudes

**DOI:** 10.3390/ijerph19095259

**Published:** 2022-04-26

**Authors:** Anna Walaszczyk, Małgorzata Koszewska, Iwona Staniec

**Affiliations:** 1 Faculty of Organization and Management, Institute of Marketing and Sustainable Development, Lodz University of Technology, Wolczanska 215 Street, 90-361 Lodz, Poland; malgorzata.koszewska@p.lodz.pl; 2 Faculty of Organization and Management, Institute of Management, Lodz University of Technology, Piotrkowska 266 Street, 90-361 Lodz, Poland; iwona.staniec@p.lodz.pl

**Keywords:** sustainable consumption, food traceability, value creation, consumer attitudes, pandemic

## Abstract

A conscious approach to the issue of food traceability on the part of consumers is essential for making rational food purchases, which in turn contributes to sustainable consumption and globally, is an element of sustainable development. The study aims to assess the changes in consumers’ buying behaviors in the context of food traceability before and during the COVID-19 pandemic, as well as the impact of sociodemographic factors on those changes. Therefore, an online survey was conducted on a sample of 1000 respondents who were Polish food consumers. The study covered aspects related to the traceability of food by consumers before and during the pandemic. The results allowed for positive verification of the H1: Polish consumers attitudes related to food buying process changed during the COVID-19 pandemic. The results didn’t allow for fully positive verification of the H2: Sociodemographic factors significantly influence Polish consumers attitudes to the food shopping during COVID-19 period compared to pre-pandemic period. The significant influence was supported in almost all (in 6 out of 8) analyzed aspects in case of age, education, and place of residence. However, in case of gender it was confirmed only in terms of two out of eight aspects: choosing product of national origin and using the online form of ordering purchases.

## 1. Introduction

Analyses and forecasts related to the COVID-19, a popular topic in 2020/2021, show that in the future, we will have to learn to live in the face of pandemics and the high variability and dynamics they generate in terms of daily functioning. Therefore, research showing, for example, how a sudden change in living conditions affects the purchasing behavior of consumers allows us to draw important lessons for the future [1,2,3,4]. The pandemic highlighted that the world needs to use its land and water resources in a sustainable manner. One of the ways to achieve this goal is to reduce food waste [5], which can be achieved or at least minimized through sustainable consumption resulting from sustainable purchasing. The COVID-19 pandemic has significantly affected many aspects of society. People around the world have given up most activities, but the need to meet life’s basic needs—such as nutrition—has not disappeared. Regardless of the place of residence in the world and the corresponding infection rate or the internal organization of a country related to the pandemic—the process of grocery shopping remained accessible.

One of the 17 Sustainable Development Goals is responsible consumption and production (Goal 12). The tasks for this goal in the 2030 Agenda for Sustainable Development include, among others, halving the global amount of food wasted per capita in retail and consumption and significantly reducing waste generation through prevention, reduction, recycling and reuse. These tasks are related to the process of food purchasing by consumers and the attitudes that accompany this process. The cumulative decisions of many consumers, as well as the decisions of individual consumers collected over the course of their entire lives, have a significant impact on the environment, even though each of these decisions taken separately would be almost meaningless [6]. The tyranny of small decisions thus applies to all consumer decisions, and in particular to: purchasing decisions (“whether to buy?”); choosing specific products or services (“what to buy?”); choosing the place of purchase (“where to buy?”); manner of use (“how to use dealing with the product at the end of its useful life (“how to throw it away?”). The implementation of the concept of sustainable development may be supported by “rational” consumption—conscious, guided by reason [7]. 

The pandemic has severely struck the global economy and the global health of populations around the world. In such a challenging time, maintaining the health and safety of workers operating food supply chains and at the same time maintaining high levels of food safety and consumer confidence is of key importance [8]. The process that plays a very important role in these conditions—which is still gaining in importance—is food traceability, a process that closely influences the quality and safety of food. The traceability of a food product is an increasingly important issue for consumers [9]. The current trend favours traceability solutions that are technologically advanced and therefore tamper-proof. For example, the blockchain solution presented by [10] is a tracking strategy that ensures reliable traceability and promotes the authenticity of supply chain operations.

The average consumer makes many dietary decisions every day [11] most of them unconsciously [12] and under the influence of cognitive and emotional problems [13]. Thus, people’s dietary decisions are influenced by several factors, including health, cost, convenience, taste [14,15] and personal and environmental safety [16]. Additional nutritional values for consumers arise from the study of animal welfare [17,18], environmental protection [19,20] and social responsibility [21]. These aspects have been noted in previous studies on sustainable consumption and, together with the value of health, they cover various dimensions in the eyes of consumers [22]. The study by Ogundijo, Tas, and Onarinde [23] found that eating and shopping habits were significantly influenced during the pandemic, as can be clearly seen when responses are classified by socio-demographic factors. These effects are confirmed by the findings of Kaya [24] on the impact of socio-economic and demographic variables on consumer knowledge and attitudes. In the context of the pandemic, the strategy of shops has also changed; it is conditioned by changes in prices and the availability of food products [25]. 

There is little research on the impact of infectious diseases on lifestyle, mental health and quality of life [26]. There is also limited research that addresses the impact of COVID-19 on consumer shopping behavior [27], especially in the context of traceability and sustainability. In order to prepare for future infectious disease or pandemic problems, it is necessary to conduct a comparative analysis before and after infectious disease outbreaks and take appropriate measures [25]. Therefore, the aim of the study is to assess the changes in consumers’ buying behaviors in the context of food traceability before and during the COVID-19 pandemic, as well as the impact of sociodemographic factors on those changes.

## 2. Literature Review and Hypothesis

### 2.1. Food Traceability in the Context of More Sustainable Consumers’ Behavior

Sustainable consumption and sustainable buying behavior can be analyzed in many different aspects. Taking into account changes caused by COVID-19 pandemic, the main challenges refer to short food supply chains, local producers, food safety and security, and sustainable rural development [28]. Considering those challenges, the aspects that refer to food traceability seem to be especially relevant. Various definitions of the term “traceability” have been provided in the food safety literature and standardization. This term appeared in 1996 in the international standard ISO 8402 on quality management and quality assurance. According to the definition contained in the standard, traceability means the possibility of tracing the history, use or location of a unit by analyzing the records that allow for its identification (PN—ISO 8402: 1996). Moe [29] entitled “Traceability Perspectives in Food Production” contains a broader definition that traceability means the ability to trace a batch of products and its history along or part of a production chain, from harvesting to transportation, storage, processing, distribution and sale, or internally—at one stage of the production chain. Schwagele [30] defines traceability through the concept of identification and states that identification enables the extraction of data from the previous stage in the chain and the provision of information to the next stage. In the opinion of L.A. Rabade and J.A. Alfaro [31], traceability means registration and tracing of processes and materials used in production. Traceability is also defined in the first international standard—ISO 22005:2007—applicable to food safety management in the context of traceability. The standard defines the following: “traceability means the ability to trace the flow of feed or food through specific stages of production, processing and distribution; the shipment may refer to the origin of the materials, the history of processing and distribution of the feed or food”.

Food traceability studies are conducted all over the world—in China [32], Japan [33] or, among others, in Brazil [34]. The conclusion of these country-specific studies or those comparing the issue of consumer perceptions of food traceability in different countries [35] is that consumers care about information such as where a product comes from and what production methods were used to create it. 

The literature review allowed us to distinguish three main aspects of consumer behavior in the context of traceability that also refer to sustainability:

- the origin of the food product [36,37,38],
- the place of shopping [28,39],
- food labels and information which they convey [40,41].

Although, those aspects were analyzed in the context of COVID-19 pandemic, but separately and never in the direct reference to food traceability. Furthermore, the aspects of the place of shopping referred mainly to the online purchases. In our study we added important aspects, omitted in the existing analyses, like: place of shopping in terms of its size and variety of supplies as well as opinion about the cleanliness.

### 2.2. The Impact of COVID-19 on Consumer Behavior and Attitude

The spread of COVID-19 worldwide has affected consumer behavior in many aspects and the impact of the pandemic on consumers’ behaviors and attitudes gained a lot of attention both in the academia and business. One of the interesting research questions worth further research is the extent to which the pandemic alters how consumers perceive and interact with the food system [36]. At the same time the pandemic highlighted the need to move to more sustainable food production and consumption patterns. Therefore, analyses that refer to changes of consumer buying behavior during COVID-19 in the context of sustainability seem to be especially important. 

We can find a lot of analyses in this area, showing the positive impact of pandemic on more sustainable buying behaviors. Globally, the Nielsen Agency conducted a study [42] on consumer attitudes and behavior during the pandemic, which found that in March 2021, there was a 33% increase in sales of certified organic food compared to the same period a year ago. Similarly, there was an increase in sales of lactose-free milk and plant-based drinks by 71% and 41%, respectively, which may indicate an increasing concern for one’s own health. A report published by McKinsey & Company (Singapore) and Euro Commerce (Etterbeek, Belgium) [43] shows that grocery sales experienced an average growth of 20% during the first lockdown in March 2020 and 10% for the whole of 2020. The same report shows that Poles have partially abandoned shopping in hypermarkets (ca. 7% drop in revenues from grocery sales) in favour of supermarkets and discount stores (increase in revenues by 16.5% and 13.6%, respectively). According to a survey conducted among Poles by KPMG during the pandemic—66% of respondents saw no reason or necessity to give up grocery shopping in brick-and-mortar shops, and the location of these shops became the most important factor for 70% of respondents when deciding where to do their shopping. The results of the same research show that the frequency of online food purchases has increased by 22%, the highest increase of any other shopping category [44]. 

The literature review showed that the research of COVID-19 impact on consumer behavior was conducted in different regions: North America [36,45], China [46,47], Australia [38] and many European countries [28,30,39,41,45,48,49,50] including Poland [51]. Most of the studies were based on quantitative research, mainly online surveys and referred to different aspects of food consumers’ behaviors. All the analyzed aspects were related to sustainability. For example: frequency and amount of food shopping, impulse, and bulk buying [30,39,45], buying healthier food [36,45], the origin and authenticity of food, local buying [36,38], food safety [36,47]. The research conducted in Poland referred only to the effects of COVID-19 on changing consumers’ eating habits, including their concerns about food service nutrition in case of new disease risk factors [52], but did not analyze the purchasing behavior in the context of traceability and sustainability.

The results of almost all reviewed papers confirmed the impact of the pandemic on the analyzed behavioral aspects. That allowed us to formulate the following hypotheses, in which we refer to the earlier identified aspects of food traceability:

**H1.**  *Polish consumers attitudes related to food buying process changed during the COVID-19 pandemic (changed in relation to the time before and during the COVID-19 pandemic).*

**H1a.**  *Polish consumers attitudes related to the **origin of the food product** (choosing product of national/local origin, not buying food from certain countries), changed during the COVID-19 pandemic.*

**H1b.**  *Polish consumers attitudes related to **the place of shopping** (paying attention to: place of shopping in terms of its size and variety of supplies, good opinion about the cleanliness, using the online form of ordering purchases) changed during the COVID-19 pandemic.*

**H1c.**  *Polish consumers attitudes related to **food labels** (reading the labels on the purchased food in terms of: manufacturer’s name/location and raw material composition of the product) changed during the COVID-19 pandemic.*

### 2.3. Determinants of Changes in Polish Consumers’ Attitudes to the Food Shopping Process during the COVID-19—The Role of Sociodemographic Characteristics

The review of the literature [28,30,36,38,39,41,42,43,44,45,46,47,49,50,51] on food consumer behavior in the context of pandemic showed that sociodemographic factors are one of the most often analyzed determinants of changes in food consumer behaviors caused by COVID-19. The impact of those factors, together with the product-related and consumer psychographics, was found to be more evident compared to supply-related factors [53].

However, the research on Czech green buying behavior modification, caused by pandemic showed that no considerable changes were seen in the context of dependence on gender, education level, or age category [41]. Similarly, Ogundijo et al. [30] research on the effects of COVID-19 on the purchasing behaviors showed no statistically important difference among the genders. However, the differences were visible in case of age as the effects of the pandemic were more noticeable in younger generations, participants in full- or part-time employment, those with higher educational qualifications, and participants from minority ethnic groups. Also, the studies made by Li et al. [47] analyzing COVID-19 lockdown effect on Chinese consumers’ purchasing and consumption behavior from a sustainability point of view, showed that gender and age are relevant factors affecting sustainable behavior.

The varied results regarding the impact of sociodemographic variables on food consumer behavior during pandemic as well as significant differences regarding consumer purchasing behaviors and preferences regarding demographic factors before and during the pandemic identified in case of wood furniture [51], allowed us to formulate the following hypothesis:

**H2.**  ***Sociodemographic factors** significantly influence Polish consumers attitudes to the food shopping process in terms of: **origin of the food product, the place of shopping and food labels** during COVID-19 period compared to pre-pandemic period.*

**H2a.**  ***Gender** significantly influence Polish consumers attitudes to the food shopping process in terms of: origin of the food product, the place of shopping and food labels.*

**H2b.**  ***Age** significantly influence Polish consumers attitudes to the food shopping process in terms of: origin of the food product, the place of shopping and food labels.*

**H2c.**  ***Education** significantly influence Polish consumers attitudes to the food shopping process in terms of: origin of the food product, the place of shopping and food labels.*

**H2d.**  ***Place of residence** significantly influence Polish consumers attitudes to the food shopping process in terms of: origin of the food product, the place of shopping and food labels.*

## 3. Materials and Methods

The research problem of the study is the impact of the pandemic on food purchasing by Polish consumers and their attitudes related to this process in the context of striving for sustainable consumption. To verify the presented hypothesis referring to the research problem posed, the survey was conducted between 11 December 2020 and 15 March 2021 among Polish consumers. The study involved 1000 randomly selected respondents in the age range of 18–80 years. The research tool was a questionnaire. The research method used was CAWI (Computer-Assisted Web Interview). The scripted questionnaire (programmed interview) was placed on the server of the company that commissioned the research, where there is a CADAS server dedicated to CAWI research, which provides access to approximately 1,800,000 panelists in Poland. In order to prepare the survey for implementation, a website link was generated and used to send survey invitations. The survey was oversampled, and quotas were set for particular characteristics (gender, age, education, place of residence). To collect 1000 effective interviews, the set amounts were about 5% higher than assumed in the project. It was possible to keep track of information on the number of completed interviews, questionnaires in progress, discontinued and rejected at the control stage (implementation control according to ESOMAR and PTBRIO standards). When one of the quotas filled up, the possibility for a person with these characteristics to complete the survey was blocked.

Participants of the study were asked to indicate, on a Likert scale [54] which attitudes they displayed as part of the purchasing process before and during the pandemic period. The five-point scale included the following answers: never (on no purchases), rarely (less frequently than every second purchase), sometimes (on average every second purchase), often (more than half of the purchases), always (with every purchase). Based on the identified aspect of the food traceability (presented in Section 2.1.) the following attitudes (the same for both analyzed periods) that refer to: the origin of the food product, the place of shopping, food labels in the questionnaire included: 

The origin of the food product:

1. choosing products of national origin,
2. choosing products of local origin,
3. not buying food from certain countries,

The place of shopping:

4. paying attention to the place of shopping in terms of its size and the related variety of supplies,
5. paying attention to the place of shopping in terms of good opinion about the shop regarding its cleanliness,
6. using the online form of ordering purchases with home delivery,

The food labels:

7. reading the labels on the purchased food in terms of the manufacturer’s name/location,
8. reading the labels on the purchased food in terms of the raw material composition of the product.

The development of a questionnaire involved the following stages: literature review and face and content validity. The initial step in developing the instrument was to identify the most representative variables. These were identified and selected based on a literature review of articles published in international journals. Content validity was assessed qualitatively. In the pilot study, experts were asked to read and evaluate the questionnaire. Ambiguities were discussed and suggested changes were made.

The justification for the selection of aspects of the study was also based on own observations of consumer attitudes in the periods studied, which resulted in the identification of attitudes that had not been studied in the conditions of the pandemic [27]. 

The collected responses of the respondents were subject to control. Among other things, the timing of the interview, as well as the consistency and logicality of the answers, were verified. After checking for completion, relationships between demographic characteristics such as gender, age, education and place of residence were analyzed. To determine whether the observed differences were statistically significant, the Bonferroni test was performed [54]. SPSS statistical software was used in the analysis of the results. The results of the study were compiled in interactive tables, and the answers to the respondents’ questions were compared with their demographic characteristics. A test developed by Frank Wilcoxon was used to assess attitudes. The Wilcoxon test [55,56] for paired observations is used to compare data collected before and during the COVID-19 pandemic to examine whether there was a statistically significant change. The test verifies the null hypothesis (H0) of the equality of median attitudes before and during the COVID-19 pandemic. It is based on the differences between the values of characteristics from the compared sets; therefore, it requires variables on an interval scale. The test has the advantage of not making assumptions about the sample distribution. 

The limitations of the research were related to the form of their implementation and scope. As far as the form of the research is concerned, the CAWI method was adopted due to the possibility of efficient research implementation in the time of a pandemic on a fairly large group of respondents. However, this method does not allow direct contact with the respondent, which limits the study in terms of making it at least partially qualitative. The scope of the study covered Poland, which, from the point of view of generalizing the results of the study, is also a research limitation (more about limitations of the research in the Discussion chapter). Identification and awareness of research limitations contribute to setting the directions for further research on the topic under consideration. Taking care of the ethical aspects of the research, its anonymity was ensured through its declaration in the introduction to the research addressed to the respondents and the lack of an option to record personal and contact details of respondents. 

## 4. Results

The structure of the respondents in terms of sex, age, education and place of residence is presented in Table 1, Table 2, Table 3 and Table 4.

The presented gender structure reflects the structure of the population in Poland, as in the surveyed age group, the share of women in the population is 51.6% (the conducted test of equality of structure indicators demonstrates that the hypothesis of equality of women’s shares in the surveyed sample and in the Polish population cannot be rejected). 

The presented age structure of the respondents, especially in the post-production age group, deviates from the structure of the population in Poland. The underrepresentation in this age group results mainly from the selected CAWI research technique. The indicator of similarity of structures in terms of age to the population of Poles designated for the surveyed group of respondents is as high as 94%, which indicates their very high similarity.

A comparative analysis of the structure of the studied sample and the population in terms of education shows their significant differences. There is an over-representation of people with higher and secondary education, which from the point of view of the aim of the survey and questions in the questionnaire is a favourable situation. However, when generalizing the results of the survey, it should be noted that they may be characteristic mainly of persons with secondary or higher education.

In general statistics, the population in Poland is not presented according to the size of the cities. There is only a division into residents of rural and urban areas, to which the characteristics in Table 4 do not apply, so it cannot be applied to the total population. Table 5 presents the descriptive statistics of the eight aspects considered in the survey of consumer attitudes in relation to food traceability in the purchasing process. The results of the study show that in all the aspects examined, the indicators showing the consumer attitude towards a given aspect during the pandemic are higher than before the pandemic. The highest increase in the indicator concerns the aspect of “not buying food from certain countries”. The smallest changes in consumer attitudes before and during the pandemic in the studied topic were observed in the context of aspects such as “choosing products of national origin” and “reading labels on the purchased food in terms of the composition of the raw product of the product”.

The analysis of Table 5 leads to the conclusion that the mean levels oscillate around the middle of the measurement scale (in the period before the pandemic they range from 2.57 to 3.38 and during the pandemic from 2.77 to 3.50) and the standard deviations oscillate around the value of one. The scatter of the data indicates a distribution that is relatively flat and, in the case of the data over time during the pandemic, an asymmetric distribution. The results indicate that the response distributions are not close to a normal distribution in the case studied. The median indicates an increase in the frequency of attitudes regarding: choosing products with national origins, choosing products with local origins, not buying food from certain countries, paying attention to the shopping location in terms of its size and associated variety of supplies, paying attention to the shopping location in terms of having a good reputation for keeping the store clean, and reading the labels on the food purchased in terms of the raw material composition of the product. In addition, 50% of respondents indicate that they implement these attitudes during the pandemic often (more often than half of their shopping) or always (every time they shop). The median also indicates identical frequencies of attitudes for using an online form of ordering groceries with home delivery and reading the labels on the foods purchased for the name/location of the manufacturer. Thus, 50% of respondents never (on no purchases), rarely (less than every other purchase), and sometimes (on average every other purchase), as part of their shopping process before and during the pandemic period, read labels on purchased foods for the manufacturer’s name/location and used the online form of ordering purchases with home delivery.

Table 6 presents a summary of consumer attitudes before and during the COVID-19 pandemic in terms of three groups: Negative Ranks (NR)—the frequency of actions in COVID-19 is lower than before, Positive Ranks (PR)—the frequency of actions in COVID-19 is greater than before, and Ties—the frequency of activities in COVID-19 is the same as before. In each of the analyzed attitudes (aspects), the frequency of actions during the pandemic was dominant—the same as before—for each of the attitudes, they constituted over 50%. In cases of attitude change, positive change was dominant (about two times higher), i.e., the frequency of actions during the pandemic was higher than before, but there were also negative changes in attitudes, i.e., the frequency of activities during the pandemic was lower than before. For all attitudes, the Wilcoxon test indicates the rejection of the null hypothesis (H0) of the equality of median attitudes before and during a pandemic in favour of the alternative hypothesis that median attitudes before the pandemic differ significantly from the median attitudes during the pandemic. In the case analyzed, all pre-pandemic attitudes are lower than during the COVID-19 pandemic.

The assessment of consumer attitudes before and during the pandemic conducted in Table 6 shows that most of the same consumer attitudes before and during the pandemic were recorded in terms of choosing products with national origin. Most positive changes i.e., frequency of actions in COVID-19 is greater than before were recorded in terms of not buying food from specific countries. The most negative changes, i.e., the frequency of actions in COVID-19 is less than before, were reported in terms of reading labels on the purchased food in terms of the raw material composition of the product.

Table 7, Table 8, Table 9 and Table 10 present the results of the survey with respect to consumer attitudes towards the examined aspects taking into account socio-demographic factors such as gender, age, education and place of residence. In Table 7, Table 8, Table 9 and Table 10, the examined aspects are presented under the numbers from 1 to 8:

1. Choosing products of national origin
2. Choosing products of local origin
3. Not buying food from certain countries
4. Paying attention to the place of shopping in terms of its size and the related variety of supplies
5. Paying attention to the place of shopping in terms of good opinion about the shop regarding its cleanliness
6. Using the online form of ordering purchases with home delivery
7. Reading the labels on the purchased food in terms of the manufacturer’s name/location
8. Reading the labels on the purchased food in terms of the raw material composition of the product

In the case of women, for all attitudes, the Wilcoxon test indicates the rejection of the null hypothesis (H0) of the equality of median attitudes before and during the pandemic in favour of the alternative hypothesis that the median attitudes before the pandemic differ significantly from the median attitudes during the pandemic. In the analyzed case, before the pandemic, the frequency indications were lower than during the COVID-19 pandemic. In the group of men, in two cases, there are no grounds to reject the null hypothesis (H0) of the equality of medians; therefore, it can be concluded that the frequency of choosing products of national origin and using the online form of ordering home-delivered purchases by men before and during the COVID pandemic 19 is identical. For attitudes (2)–(5), (7), (8) in the male group, the null hypothesis (H0) of equality of median attitudes before and during the pandemic is rejected in favor of the alternative hypothesis that median attitudes before the pandemic are significantly different from median attitudes during the pandemic.

The results in Table 7 indicate that there is no gender influence on Polish consumers’ approach to the food shopping process in terms of (2) choosing locally sourced products, (3) not buying food from specific countries, (4) paying attention to the place of shopping in terms of its size and the associated variety of supplies (5) paying attention to the shopping location in terms of the store’s good reputation for keeping the store clean, (7) reading the labels on the foods purchased in terms of the manufacturer’s name/location, (8) reading the labels on the foods purchased in terms of the raw material composition of the product in relation to the time before and during the COVID-19 pandemic. In contrast, men and women differ significantly in attitudes regarding (1) choosing products with domestic origins and (6) using an online form of ordering groceries with home delivery. Men’s attitudes did not change in this regard with respect to before and during COVID-19 pandemic, while women’s attitudes on actions (1) and (6) increased in frequency during COVID-19 compared to before the pandemic.

Individual age groups show differentiation in terms of changes in consumer attitudes. In the 18–24 age group, the frequency of not buying food from certain countries and paying attention to the place of shopping in terms of a good opinion about the shop regarding its cleanliness has significantly changed (increased) in comparison to the time before and during the pandemic. Other attitudes have not changed. In the 25–34 age group, there has been a more significant change (increase) in the frequency of choosing products of local origin, not buying food from specific countries, paying attention to the place of shopping in terms of good opinion about the shop regarding its cleanliness and using the online form of ordering purchases with home delivery. Other attitudes have not changed. In the 35–44 age group, there has been a more significant change (increase) in the frequency of not buying food from specific countries, paying attention to the place of shopping in terms of its size and the related variety of supplies, paying attention to the place of shopping in terms of good opinion about the shop regarding its cleanliness clean and using the online form of ordering purchases with home delivery. Other attitudes have not changed. In the 45–54 age group, as in the 55–64 age group—all attitudes changed significantly. In the 65–80 age group, there was a more significant change (increase) in the frequency of choosing products of local origin, not buying food from specific countries, paying attention to the place of shopping in terms of good opinion about the shop regarding its cleanliness and reading the labels on purchased food in terms of the name/location of the producer. 

In summary, the age group of the respondents significantly affects the approach frequency of activities (1), (2), (4), (6), (7), (8) in the COVID-19 period compared to the pre-pandemic period. In contrast, age group of the respondent does not affect approach frequency (3) and (5) during COVID-19 and pre-pandemic period.

Respondents with lower secondary education and below show no significant change in the frequency of choosing products of national and local origin, not buying food from specific countries, paying attention to the place of shopping in terms of a good opinion about the shop regarding its cleanliness, using the Internet forms of ordering purchases with home delivery, reading the labels on the purchased food in terms of the name/location of the producer and reading the labels on the purchased food in terms of the raw material composition of the product. Respondents with vocational education show no significant change in the frequency of choosing products of national origin, paying attention to the place of shopping in terms of good opinion about the shop regarding its cleanliness, using the online form of ordering home delivery, reading labels on the purchased food in terms of the name/location of the producer and reading the labels on the purchased food in terms of the raw material composition of the product. Respondents with secondary education show no significant change only in the frequency of choosing products of domestic origin. Respondents with higher education show significant changes from before and during the pandemic towards all attitudes.

In summary, the respondent’s education significantly affects the approach frequency of activities (1), (2), (3), (5), (6), (7), (8) during COVID-19 period compared to pre-pandemic period. In contrast, respondent’s education does not affect approach frequency (4) in COVID-19 period and pre-pandemic period.

Respondents living in the countryside or town up to 5000 residents show no significant change in the frequency of using the online form of ordering home delivery purchases. Respondents living in a city of 5000–49,000 residents show significant changes towards all attitudes. Respondents living in a city of 50,000–199,000 residents show no significant change in attitudes in the frequency of choosing products of national origin, paying attention to the place of shopping in terms of its size and the related variety of supplies and reading the labels on the purchased food in terms of the name/location of the producer. Respondents living in a city of 200,000–499,000 residents show no significant change in the frequency of choosing products of national origin, choosing products of local origin and reading the labels on the purchased food in terms of the raw material composition of the product. Respondents living in a city with more than 500,000 residents show no significant change in the frequency of choosing products of national origin, paying attention to the place of shopping in terms of its size and the related variety of supplies and reading the labels on the purchased food in terms of the raw material composition of the product.

In conclusion, the approach of Polish consumers to the implementation of the food shopping process in (1), (2), (4), (6), (7), (8) in relation to the time before and during the COVID-19 pandemic is significantly influenced by the place of residence. On the other hand, the respondent’s place of residence does not affect Polish consumers’ attitude towards the implementation of food shopping process in terms of (3) and (5) with respect to the time before and during COVID-19 pandemic.

The presented research results allowed for the verification of the assumed research hypotheses. A summary regarding H1 and H2 is presented in Table 11 and Table 12.

The results allowed for positive verification of H1: “*Polish consumers attitudes related to food buying process changed in relation to the time before and during the COVID-19 pandemic in all analyzed aspects: origin of the food product, the place of shopping and food labels*”.

The results didn’t allow for fully positive verification of the H2: “*Sociodemographic factors significantly influence Polish consumers attitudes to the food shopping process in terms of: origin of the food product, the place of shopping and food labels during COVID-19 period compared to pre-pandemic period*” in all analyzed aspects. The significant influence on Polish consumers attitudes to the food shopping process was supported in almost all (in 6 out of 8) the aspects in case of age, education and place of residence. However, in case of gender it was confirmed only in terms of two out of eight aspects: choosing product of national origin and using the online form of ordering purchases.

## 5. Discussion

Summarizing the conducted consumer research, on the basis of the collected quantitative data, it can be concluded that the greatest change in the context of consumer attitudes (before and during the pandemic) is observed with regard to not buying food from specific countries. 

For all the studied aspects of food traceability, median consumer attitudes during the pandemic are higher than before the pandemic. This means that, to a varying degree (sometimes minimal), the level of choice of products of local and national origin by Polish consumers has increased, consumers pay more attention to the place of shopping in terms of store size, supplies, good reputation, and cleanliness. The frequency of using the online form of ordering food with home delivery has also increased, and consumers read food labels more carefully, both in terms of raw material composition and the name/location of the producer. 

Therefore, we can conclude that the pandemic has changed consumer attitudes towards the process of purchasing food in all examined aspects. Those results allowed to confirm the H1 and are consistent with earlier studies, that analyzed the impact of COVID-19 pandemic on changes in food consumer behaviors [28,36,38,45].

The aim of the study was also to assess the impact of sociodemographic factors on the changes in consumers’ buying behaviors in the context of food traceability before and during the COVID-19 pandemic. In this case the results are more complex and ambiguous. They indicate that there is no gender influence on Polish consumers’ approach to the food shopping process in case of most of analyzed aspects: choosing local product and avoiding food form specific countries, paying attention to the place of shopping and those related to the labels. These results are in line with those reported in [30,41]. 

However, in two other aspects: choosing products with domestic origins and online form of ordering differences between men and women were identified. It can therefore be concluded that gender differentiates consumers behaviors, but only in specific areas of food purchasing process.

In case of other analyzed sociodemographic characteristic such as: education, age and the place of residence the results confirmed their significant influence is case of the vast majority of the analyzed aspects of the food shopping process.

These results yielded support for past previous studies, where age and education significantly influenced effects of COVID-19 on the purchasing behaviors [30,47].

As the review of the literature on the issues discussed in the article showed—many studies have been conducted worldwide in the context of changes in consumer behavior in the pandemic era. However, with regard to the aspect of food traceability, these studies are relatively scarce. The results of the study presented in this article have identified limitations. These limitations include the CAWI research technique used and the area of the study limited to Poland. The applied research technique limited the availability of the study for rural residents, people with education below lower secondary school, and respondents in post-working age. Conclusions from the conducted research constitute the basis for determining several key implications, both of a social and economic/business nature. First, from a macro-economic point of view, it is worth taking measures to develop local and national products (the study showed that during the pandemic, consumers were more likely to buy these products than before the pandemic and avoided products from certain countries). Second, consumer awareness of the cleanliness requirements of shops has increased during the pandemic. This situation may make it necessary for retailers/store owners to adapt to higher standards of cleanliness in food retail premises. Third, manufacturers should pay attention to the accuracy of information provided on product labels, as the pandemic has increased consumer interest in this area.

## 6. Conclusions

The survey results suggest that changes in consumer attitudes will affect food traceability and that consumers will demand the market to introduce technologically advanced and tamper-proof solutions in this regard. A reliably conducted traceability process, apart from the obvious fact of providing the consumer with information about the product “from farm to fork”, also allows for increased supervision of food safety (throughout the entire traceability process), its quantity on the market (so-called forward traceability) and the way it is produced (so-called backward traceability). The process supervision in question provides quantifiable information for making decisions aimed at achieving sustainable development in the context of sustainable production and sustainable consumption (indirectly sustainable purchasing).

Sustainable development expectations of consumers and other stakeholders require organizations to integrate sustainability into their entire supply chain, shifting their focus from traditional profit-seeking to addressing environmental and social sustainability problems [57,58,59,60,61]. The coronavirus pandemic has exposed many things, and none is more evident than how interconnected our world is. The impact of globalization is most apparent in the jamming supply chains that threaten food security around the world. Maintaining or re-weaving these networks will require technology, innovation and political determination [4].

## Figures and Tables

**Table 1:** Gender of the respondents.

GENDER	Frequency	%	Cumulative %	% of the Population
woman	526	52.6	52.6	51.6
man	474	47.4	100	48.4
in total	1000	100		

Source: own study based on own research.

**Table 2:** Age of the respondents.

AGE	Frequency	%	Cumulative %	% of the Population	Similarity Index
18–24	99	9.9	9.9	9.2	9.2
25–34	194	19.4	29.3	18.8	18.8
35–44	205	20.5	49.8	20.2	20.2
45–54	161	16.1	65.9	13.6	13.6
55–64	169	16.9	82.8	15.0	15.0
65–80	172	17.2	100	23.2	17.2
in total	1000	100		100	94

Source: own study based on own research.

**Table 3:** Education of the respondents.

EDUCATION	Frequency	%	Cumulative %	% of the Population	Similarity Index
junior high school or lower	13	1.3	1.3	30.7	1.3
essential vocational	94	9.4	10.7	23.3	10.7
secondary/secondary vocational/post-secondary	454	45.4	56.1	33	33
higher	439	43.9	100	13	13
in total	1000	100		100	58

Source: own study based on own research.

**Table 4:** Place of residence of the respondents.

PLACE OF THE RESIDENCE	Frequency	%	Cumulative %
village or city up to 5000	306	30.6	30.6
city of 5000–49,000	262	26.2	56.8
city of 50,000–199,000	172	17.2	74.0
city of 200,000–499,000	98	9.8	83.8
city over 500,000	162	16.2	100
in total	1000	100	

Source: own study based on own research.

**Table 5:** Descriptive statistics of the examined aspects.

	before the Pandemic			during the Pandemic		
	Mean	Std. Deviation	Median	Mean	Std. Deviation	Median
Choosing products of national origin	3.38	1.085	3.00	3.49	1.063	4.00
Choosing products of local origin	3.26	1.092	3.00	3.42	1.073	4.00
Not buying food from certain countries	2.57	1.254	2.00	2.93	1.247	3.00
Paying attention to the place of shopping in terms of its size and the related variety of supplies	3.34	1.128	3.00	3.53	1.064	4.00
Paying attention to the place of shopping in terms of good opinion about the shop regarding its cleanliness	3.26	1.209	3.00	3.48	1.154	4.00
Using the online form of ordering purchases with home delivery	2.61	1.226	3.00	2.77	1.254	3.00
Reading the labels on the purchased food in terms of the manufacturer’s name/location	3.15	1.170	3.00	3.32	1.150	3.00
Reading the labels on the purchased food in terms of the raw material composition of the product	3.38	1.121	3.00	3.50	1.107	4.00

Source: own study based on own research.

**Table 6:** Assessment of consumer attitudes before and during the pandemic.

		N	Mean Rank	Sum of Ranks	Z	Asymp. Sig. (2-Tailed)
Choosing products of national origin	NR	158	190.16	30,045.00	−4.116	<0.0001
	PR	235	201.60	47,376,00		
	Ties	607				
Choosing products of local origin	NR	145	197.43	28,628.00	−5.93	<0.0001
	PR	264	209.16	55,217.00		
	Ties	591				
Not buying food from certain countries	NR	116	183.61	21,299.00	−10.927	<0.0001
	PR	329	236.89	77,936.00		
	Ties	555				
Paying attention to the place of shopping in terms of its size and the related variety of supplies	NR	164	211.00	34,604.00	−5.74	<0.0001
	PR	280	229.24	64,186.00		
	Ties	556				
Paying attention to the place of shopping in terms of good opinion about the shop regarding its cleanliness	NR	138	202.00	27,876.50	−7063	<0.0001
	PR	284	216.11	61,376.50		
	Ties	578				
Using the online form of ordering purchases with home delivery	NR	159	203.49	32,355.50	−5423	<0.0001
	PR	267	219.46	58,595.50		
	Ties	574				
Reading the labels on the purchased food in terms of the manufacturer’s name/location	NR	164	224.73	36,856.50	−5379	<0.0001
	PR	287	226.72	65,069.50		
	Ties	549				
Reading the labels on the purchased food in terms of the raw material composition of the product	NR	166	206.95	34,353.50	−4.29	<0.0001
	PR	255	213.64	54,477.50		
	Ties	579				

NR—Negative Ranks- the incidence of activities during COVID-19 is less than before. PR—Positive Ranks- the frequency of activities during COVID-19 is greater than before. Ties—the frequency of activities during COVID-19 is the same as before. Source: own study based on own research.

**Table 7:** Gender-based consumer attitudes.

		1	2	3	4	5	6	7	8
**woman**	Z	−4.447	−5.211	−8.238	−5.098	−5.614	−5.49	−4.397	−3.505
	Asymp. Sig. (2-tailed)	<0.0001	<0.0001	<0.0001	<0.0001	<0.0001	<0.0001	<0.0001	<0.0001
**man**	Z	−1.116	−3.048	−7.172	−2.857	−4.328	−1.67	−3.143	−2.509
	Asymp. Sig. (2-tailed)	0.264	0.002	<0.0001	0.004	<0.0001	0.095	0.002	0.012

grey marking means that the pandemic did not affect consumer attitudes *p* > 0.05. Source: own study based on own research.

**Table 8:** Age-based consumer attitudes.

		1	2	3	4	5	6	7	8
**18**–**24**	Z	−1.232	−0.459	−2.656	−0.954	−2.498	−0.702	−0.919	−1.029
	Asymp. Sig. (2-tailed)	0.218	0.646	0.008	0.340	0.012	0.483	0.358	0.304
**25**–**34**	Z	−1.527	−3.141	−5.250	−1.591	−2.805	−3.246	−1.284	−0.788
	Asymp. Sig. (2-tailed)	0.127	0.002	<0.0001	0.112	0.005	0.001	0.199	0.431
**35**–**44**	Z	−1.417	−1.766	−4.603	−4.667	−2.946	−3.371	−1.026	−1.327
	Asymp. Sig. (2-tailed)	0.157	0.077	<0.0001	<0.0001	0.003	0.001	0.305	0.185
**45**–**54**	Z	−4.127	−2.917	−4.686	−2.496	−2.683	−1.967	−2.540	−2.967
	Asymp. Sig. (2-tailed)	<0.0001	0.004	<0.0001	0.013	0.007	0.049	0.011	0.003
**55**–**64**	Z	−2.003	−3.078	−4.366	−2.358	−3.618	−2.930	−3.528	−2.742
	Asymp. Sig. (2-tailed)	0.045	0.002	0.000	0.018	<0.0001	0.003	<0.0001	0.006
**65**–**80**	Z	−0.364	−2.811	−4.888	−1.538	−2.840	−0.541	−3.967	−1.796
	Asymp. Sig. (2-tailed)	0.716	0.005	<0.0001	0.124	0.005	0.589	<0.0001	0.072

grey marking means that the pandemic did not affect consumer attitudes *p* > 0.05. Source: own study based on own research.

**Table 9:** Education-based consumer attitudes.

		1	2	3	4	5	6	7	8
**junior** **high** **school or lower**	Z	−1.242	−0.425	−1.725	−2.126	−0.368	−0.368	−0.979	−0.957
	Asymp. Sig. (2-tailed)	0.214	0.671	0.084	0.033	0.713	0.713	0.327	0.339
**essential vocational**	Z	−1.252	−2.042	−3.628	−2.186	−0.303	−0.599	−0.487	−0.195
	Asymp. Sig. (2-tailed)	0.210	0.041	<0.0001	0.029	0.762	0.549	0.626	0.845
**secondary/secondary vocational/post-secondary**	Z	−1.165	−2.121	−6.311	−2744	−4.531	−3.758	−3.609	−3.202
	Asymp. Sig. (2-tailed)	0.244	0.034	<0.0001	0.006	<0.0001	<0.0001	<0.0001	0.001
**higher**	Z	−4.157	−5.718	−8.02	−4.387	−6.065	−4.802	−4.027	−2.951
	Asymp. Sig. (2-tailed)	<0.0001	<0.0001	<0.0001	<0.0001	<0.0001	<0.0001	<0.0001	0.003

grey marking means that the pandemic did not affect consumer attitudes *p* > 0.05. Source: own study based on own research.

**Table 10:** Place of residence-based consumer attitudes.

		1	2	3	4	5	6	7	8
village or city up to 5000	Z	−2.567	−4.386	−7.867	−3.534	−3.322	−0.912	−3.701	−3.166
	Asymp. Sig. (2-tailed)	0.010	<0.0001	<0.0001	<0.0001	0.001	0.362	<0.0001	0.002
city of 5000–49,000	Z	−2.775	−2.639	−4.576	−4.247	−4.619	−4.538	−2.768	−2.154
	Asymp. Sig. (2-tailed)	0.006	0.008	0.000	0.000	0.000	0.000	0.006	0.031
city of 50,000–199,000	Z	−1.272	−1.975	−3.563	−1.423	−2.442	−2.368	−1.067	−2.084
	Asymp. Sig. (2-tailed)	0.203	0.048	<0.0001	0.155	0.015	0.018	0.286	0.037
city of 200,000– 499,000	Z	−1.132	−1.345	−2.818	−2.113	−1.997	−2.078	−2.257	−1.47
	Asymp. Sig. (2-tailed)	0.258	0.179	0.005	0.035	0.046	0.038	0.024	0.142
city over 500,000	Z	−0.966	−2.153	−4.458	−0.892	−3.016	−2.163	−2.032	−0.098
	Asymp. Sig. (2-tailed)	0.334	0.031	<0.0001	0.372	0.003	0.031	0.042	0.922

grey marking means that the pandemic did not affect consumer attitudes *p* > 0.05. Source: own study based on own research.

**Table 11:** Verification of the Hypothesis H1.

H1	Polish Consumers Attitudes Related to Food Buying Process Changed during the COVID-19 Pandemic (Changed in Relation to the Time before and during the COVID-19 Pandemic)	Positively Verified		
H1a	Polish consumers attitudes related to the origin of the food product; (1) choosing product of national origin (2) choosing product of local origin (3) not buying food from certain countries, changed during the COVID-19 pandemic	*p* < 0.0001 *p* < 0.0001 *p* < 0.0001	supported supported supported	positively verified
H1b	Polish consumers attitudes related to the place of shopping (1) paying attention to place of shopping in terms of its size and variety of supplies, (2) paying attention to good opinion about the cleanliness, (3) using the online form of ordering purchases changed during the COVID-19 pandemic	*p* < 0.0001 *p* < 0.0001 *p* < 0.0001	supported supported supported	positively verified
H1c	Polish consumers attitudes related to food labels—reading the labels on the purchased food in terms of (1) manufacturer’s name/location (2) raw material composition of the product changed during the COVID-19 pandemic	*p* < 0.0001 *p* < 0.0001	supported supported	positively verified

Source: own study based on own research.

**Table 12:** Verification of the Hypothesis H2.

H2	Socio-Demographic Factors Significantly Influence Polish Consumers Attitudes to the Food Shopping Process in Terms of: Origin of the Food Product, the Place of Shopping and Food Labels during COVID-19 Period Compared to Pre-Pandemic Period		
H2a	Gender significantly influence Polish consumers attitudes to the food shopping process in terms of:		
	(1) choosing product of national origin (2) choosing product of local origin (3) not buying food from certain countries, (4) paying attention to place of shopping in terms of its size and variety of supplies, (5) paying attention to:;good opinion about the cleanliness, (6) using the online form of ordering purchases (7) manufacturer’s name/location (8) raw material composition of the product	*p* < 0.05 *p* > 0.05 *p* > 0.05 *p* > 0.05 *p* > 0.05 *p* < 0.05 *p* > 0.05 *p* > 0.05	supported not supported not supported not supported not supported supported not supported not supported
H2b	Age significantly influence Polish consumers attitudes to the food shopping process in terms of:		
	(1) choosing product of national origin (2) choosing product of local origi (3) nnot buying food from certain countries, (4) paying attention to place of shopping in terms of its size and variety of supplies, (5) paying attention to:;good opinion about the cleanliness, (6) using the online form of ordering purchases (7) manufacturer’s name/location (8) raw material composition of the product	*p* < 0.05 *p* < 0.05 *p* > 0.05 *p* < 0.05 *p* < 0.05 *p* > 0.05 *p* < 0.05 *p* < 0.05	supported supported not supported supported supported not supported supported supported
H2c	Education significantly influence Polish consumers attitudes to the food shopping process in terms of:		
	(1) choosing product of national origin (2) choosing product of local origin (3) not buying food from certain countries, (4) paying attention to place of shopping in terms of its size and variety of supplies, (5) paying attention to:;good opinion about the cleanliness, (6) using the online form of ordering purchases (7) manufacturer’s name/location (8) raw material composition of the product	*p* < 0.05 *p* < 0.05 *p* < 0.05 *p* > 0.05 *p* < 0.05 *p* < 0.05 *p* < 0.05 *p* < 0.05	supported supported supported not supported supported supported supported supported
H2d	Place of residence significantly influence Polish consumers attitudes to the food shopping process in terms of:		
	(1) choosing product of national origin (2) choosing product of local origin (3) not buying food from certain countries, (4) paying attention to place of shopping in terms of its size and variety of supplies, (5) paying attention to good opinion about the cleanliness, (6) using the online form of ordering purchases (7) manufacturer’s name/location (8) raw material composition of the product	*p* < 0.05 *p* < 0.05 *p* > 0.05 *p* < 0.05 *p* < 0.05 *p* > 0.05 *p* < 0.05 *p* < 0.05	supported supported not supported supported supported not supported supported supported

Source: own study based on own research.

## Data Availability

The data presented in this study are available on request from the corresponding author.
